# Timing of therapeutic hypothermia and outcomes in neonates with hypoxic-ischemic encephalopathy: A cohort study in a middle-income country

**DOI:** 10.1371/journal.pone.0343589

**Published:** 2026-03-09

**Authors:** Sergio Agudelo-Pérez, Gloria Troncoso, Lina María Gutiérrez Montenegro, Juliana López Ordoñez

**Affiliations:** 1 Department of Pediatrics, School of Medicine, Universidad de La Sabana, Chía, Colombia; 2 Neonatal Unit, Fundación Cardio Infantil – Instituto de Cardiología, Bogotá, Colombia; 3 School of Medicine, Universidad de La Sabana, Chía, Colombia; UNBC: University of Northern British Columbia, CANADA

## Abstract

**Background:**

Therapeutic hypothermia improves survival and neurodevelopmental outcomes in neonates with hypoxic-ischemic encephalopathy when initiated within 6 hours of birth. However, in low- and middle-income countries, delays in referral and access to tertiary care often preclude early initiation and the benefits of therapeutic hypothermia beyond the recommended window remain uncertain. We aimed to assess whether initiating therapeutic hypothermia between 6 and 12 hours after birth is associated with a higher risk of mortality and/or brain injury than initiation within 6 hours in neonates with moderate or severe hypoxic-ischemic encephalopathy.

**Methods:**

We conducted a retrospective cohort study of 173 neonates with moderate or severe hypoxic-ischemic encephalopathy treated with servo-controlled whole-body therapeutic hypothermia at a tertiary care center in Colombia. Neonates were categorized based on the timing of therapeutic hypothermia initiation as ≤6 h or >6–12 h after birth. The primary outcome was a composite of in-hospital mortality and/or brain injury confirmed by magnetic resonance imaging during the first week of life. Multivariate logistic regression was used to adjust for confounding variables.

**Results:**

Of the 173 neonates, 44.5% received therapeutic hypothermia within 6 hours and 55.5% after 6–12 hours. A composite outcome was observed in 40.6% of the patients. Delayed therapeutic hypothermia was not significantly associated with an increased risk of the composite outcome compared to early initiation (adjusted odds ratio [OR]: 1.83; 95% CI: 0.86–3.90). Seizures and severe hypoxic-ischemic encephalopathy were found to be independent predictors of adverse outcomes.

**Conclusions:**

In this cohort, initiation of therapeutic hypothermia between 6 and 12 h after birth was not significantly associated with worse neurological or mortality outcomes than initiation within 6 h. These findings suggest that delayed therapeutic hypothermia may still confer benefits in settings where early initiation is challenging, underscoring the need to strengthen referral systems and further investigate the optimal therapeutic window.

## Introduction

Perinatal asphyxia is a major contributor to neonatal mortality and long-term disabilities [1,2]. The prevalence of hypoxic-ischemic encephalopathy (HIE) in low- and middle-income countries ranges from 15.9% to 35% [3,4]. Surviving neonates face an eight-fold increased risk of developing neurological sequelae, particularly in settings with limited access to neuroprotective interventions [5].

Therapeutic hypothermia (TH) remains the only intervention with robust evidence supporting its neuroprotective efficacy in neonates with perinatal asphyxia [6,7]. TH mitigates secondary brain injury by suppressing metabolic and inflammatory response cascades [8]. The recommendation to initiate TH within six hours is primarily based on preclinical animal models, which demonstrate that early cooling reduces neuronal injury and improves neurological outcome [9,10]. These results prompted pivotal clinical trials (CoolCap, NICHD, and TOBY), which established a six-hour threshold and influenced international guidelines [11–13].

However, the exact timing of irreversible brain injury in HIE remains unclear and emerging pathophysiological evidence suggests that a rigid temporal threshold may not be universally optimal [14]. Animal models have indicated that the duration of the latent phase may vary depending on the severity of the insult, which potentially explains the benefits of earlier interventions [15].

In clinical practice, the effectiveness of TH is influenced not only by biological variables such as systemic inflammation, nutritional status, and genetic susceptibility [15,16], but also by contextual factors. In LMICs, structural and logistical barriers often delay the recognition, stabilization, and transport of affected neonates. As a result, many infants do not reach tertiary centers within the recommended six-hour window [17–19]. Indeed, delayed arrival is one of the most common reasons for not initiating cooling despite clinical eligibility [18], and mortality has been reported to be significantly higher in non-cooled infants than in those receiving TH [18].

Given these realities, it is essential to examine whether a broader therapeutic window, extending up to 12 hours of life, might still offer neuroprotective benefits, particularly in moderate cases of HIE. Such an approach could represent a pragmatic strategy to bridge the gap between evidence-based recommendations and real-world constraints in LMICs.

Recent studies have suggested that TH initiated after six hours may still be beneficial in selected cases [16], and some clinical guidelines now acknowledge this possibility [20,21]. However, robust data supporting delayed TH are scarce, particularly in resource-limited settings.

This study aimed to assess the association between standard TH initiation (≤6 hours) and delayed TH initiation (>6 to ≤12 hours) with in-hospital mortality and brain injury detected by MRI during the first week of life in neonates with moderate to severe perinatal asphyxia, referred to as a high-complexity center in a middle-income country. We hypothesized that no significant differences in outcomes would be observed based on the timing of the TH initiation.

## Materials and methods

This retrospective cohort study included neonates who received servo-controlled therapeutic hypothermia (TH) and were referred to a high-complexity healthcare institution in a middle- to low-income country between January 2021 and November 2022. The study protocol was approved by the Institutional Research Ethics Committee (CEIC-0474–2023). Given the retrospective nature of this study, the requirement for informed consent was waived. We accessed the data between January 5, 2024, and August 31, 2024. The authors had access to identifying information during data collection; however, the data were anonymized before analysis.

Eligible participants included term infants (>37 weeks’ gestational age, determined by the Ballard scoring system) and late preterm neonates (≥35 weeks) with a postnatal age of ≤12 h at admission, a diagnosis of moderate or severe perinatal asphyxia, and fulfillment of the institutional criteria for TH.

Moderate or severe perinatal asphyxia was defined by umbilical cord blood gas analysis (pH ≤ 7.0 and/or base deficit ≤−16) or postnatal blood gas within the first hour of life (pH between 7.01 and 7.15 and/or base deficit −10 to −15.9). Additionally, at least one of the following criteria was required: (1) acute or sentinel perinatal event, (2) Apgar score ≤5 at 10 minutes, or (3) moderate-to-severe hypoxic-ischemic encephalopathy (Sarnat stage 2 or 3). Exclusion criteria were intrauterine growth restriction (birth weight <1800 g), congenital anomalies, central nervous system malformations, congenital heart disease, and chromosomal abnormalities.

All neonates received servo-controlled whole-body therapeutic hypothermia, according to an institutional protocol based on international guidelines. Cooling was initiated in the neonatal intensive care unit by using a neonatal Thermowrap system with continuous rectal temperature monitoring. A 40-minute induction phase was used to achieve a core temperature of 33–34°C, followed by 72 hours of maintenance at 33.5 ± 0.5°C, and gradual rewarming to 36.5°C over a minimum of six hours. Conventional therapeutic hypothermia was defined as initiation within the first 6 hours of life, whereas late-onset therapeutic hypothermia was defined as initiation between >6 and ≤12 hours of life.

The primary outcome was a composite of in-hospital mortality and/or brain injury detected using magnetic resonance imaging (MRI) within the first week of life. Brain MRI was performed according to a standardized institutional protocol and was routinely scheduled around the seventh day of life for all eligible neonates regardless of the timing of therapeutic hypothermia initiation. MRI was performed using a Philips Achieva dStream system and included T1- and T2-weighted spin-echo sequences, diffusion-weighted imaging (DWI), and apparent diffusion coefficient (ADC) maps. MRI abnormalities were classified according to their anatomical location according to the framework established by the national consensus [21]: (1) lesions involving the basal ganglia and thalami, (2) cortical and watershed regions, (3) subcortical white matter, (4) periventricular white matter, (5) vascular territories (arterial infarcts), and (6) venous system (sinovenous thrombosis). The presence of at least one of these criteria can lead to abnormal brain MRI findings.

### Statistical analysis

The sample size estimation was based on the event per variable (EPV) principle for multivariable logistic regression, as proposed by Peduzzi et al. This guideline recommends a minimum of 10 outcome events per predictor variable to reduce overfitting and improve the stability of coefficient estimates. Assuming an expected prevalence of abnormal brain MRI findings of 39.3% in neonates with hypoxic-ischemic encephalopathy (HIE) treated with therapeutic hypothermia [17] and planning to include six independent variables in the model, a minimum of 173 subjects were calculated to ensure at least 10 events per variable, thus satisfying the EPV criterion.

Descriptive statistics were used to summarize baseline and clinical characteristics. Continuous variables are presented as means with standard deviations or medians with interquartile ranges, depending on their distribution assessed using the Shapiro–Wilk test. Categorical variables were expressed as absolute frequencies and percentages.

Comparisons between groups were performed using independent-samples Student’s t-test or Mann–Whitney U test for continuous variables and Chi-square or Fisher’s exact test for categorical variables, as appropriate. The assumptions for these tests, including normality, were verified prior to analysis. Outliers were explored, but they did not substantially impact the results; therefore, all the observations were retained. No transformation was applied to the data. Missing data were minimal and limited to secondary analysis, excluding neonates who died before MRI; otherwise, complete case analysis was performed.

Multivariate logistic regression models were constructed to assess the association between the timing of therapeutic hypothermia (≤6 h vs. > 6– ≤ 12 h) and the primary composite outcome of in-hospital mortality and/or MRI-confirmed brain injury. Covariates were selected a priori based on biological plausibility and retained if they met a univariate screening threshold of P < 0.25. The initial full model included six prespecified covariates plus the main explanatory variable (TH initiation time): admission temperature category (<33°C, 33–35°C, > 35°C), gestational age (≥37 vs. 35–37 weeks), severity of encephalopathy (moderate vs. severe, based on Sarnat staging), severity of asphyxia (moderate vs. severe), and presence of electrographic or clinical seizures (no vs. yes).

Backward stepwise selection (exit criterion, p > 0.10) was used to enhance parsimony, while maintaining robustness. The final adjusted model retained variables that contributed meaningfully to model fit and confounding control, as assessed by information criteria and clinical relevance, to reduce overfitting and improve interpretability. As this was a retrospective cohort study, the model included key variables associated with both the likelihood of earlier hypothermia and the risk of adverse outcomes, particularly HIE severity and seizures, to reduce confounding by indication. An interaction between the timing of TH and HIE severity was formally tested but was not statistically significant and therefore not retained in the final model.

Assumptions of the logistic regression models were assessed, including the linearity of continuous predictors in the logit model and absence of multicollinearity. Collinearity was evaluated using variance inflation factors and no significant collinearity was detected. Potential interactions between covariates were also explored and found to be non-significant. Model performance was evaluated using McFadden’s pseudo-R^2^, Akaike Information Criterion (AIC), Bayesian Information Criterion (BIC), and Hosmer–Lemeshow goodness-of-fit test.

A secondary analysis was performed using isolated brain injury on MRI as the outcome, excluding patients who died before the imaging (n = 11). This approach aims to reduce heterogeneity in the composite outcome and allows for a more homogeneous and objective assessment of neurological injury.

The results were presented as crude and adjusted odds ratios (OR and aOR) with 95% confidence intervals. Statistical significance was set at a two-tailed p-value of <0.05. All analyses were performed using RStudio (version 2025.05.0; Build 496; Posit Software, PBC, USA).

## Results

A total of 173 neonates were included in the analysis, of which 44.5% received therapeutic hypothermia (TH) within the first 6 h of life. Baseline characteristics were generally comparable between the groups, except for a significantly higher proportion of severe hypoxic-ischemic encephalopathy (HIE) among neonates who received early TH (p < 0.001) (Table 1).

**Table 1:** Clinical characteristics of the study population according to timing of therapeutic hypothermia initiation (≤6 h vs > 6–12 h).

Variable	Total (n = 173)		TH ≤ 6 hours (n = 77)		TH > 6 - ≤ 12 hours (n = 96)		P-value
Gestational age (Ballard), n (%)							
≥37 weeks	153	(88.4)	71	(46.4)	82	(53.6)	0.16
35 - 37 weeks	20	(11.6)	6	(30.0)	14	(70.0)	
Birth weight (g), n (%)**							
≥2500	150	(86.7)	68	(45.3)	82	(54.7)	0.57
<2500	23	(13.3)	9	(39.1)	14	(60.9)	
Sex, n (%)							
Female	66	(38.2)	30	(45.5)	36	(54.5)	0.84
Male	107	(61.8)	47	(43.9)	60	(56.1)	
Admission temperature (°C), n (%)							
33 - 35	97	(56.1)	47	(48.5)	50	(51.5)	0.08
<33	28	(16.2)	15	(53.6)	13	(46.4)	
>35	48	(27.7)	15	(31.3)	33	(68.7)	
Birth resuscitation, n (%)							
No	16	(9.2)	9	(56.3)	7	(43.7)	0.32
Yes	157	(90.8)	68	(43.3)	89	(56.7)	
Severity encephalopathy (Sarnat), n (%)							
Moderate	114	(65.9)	35	(30.7)	79	(69.3)	**<0.001**
Severe	59	(34.1)	42	(71.2)	17	(28.8)	
Asphyxia severity, n (%)							
Moderate	71	(41.0)	36	(50.7)	35	(49.3)	0.17
Severe	102	(59.0)	41	(40.2)	61	(59.8)	
APGAR 5 minutes, n (%)							
>5	87	(50.3)	45	(51.7)	42	(48.3)	0.06
≤5	86	(49.7)	32	(37.2)	54	(62.8)	
APGAR 10 minutes, n (%)							
>5	158	(91.3)	70	(44.3)	88	(55.7)	0.86
≤5	15	(8.7)	7	(46.7)	8	(53.3)	
Seizures, n (%)							
No	95	(60.1)	39	(41.1)	56	(58.9)	0.11
Yes	63	(39.9)	34	(54.0)	29	(46.0)	
Inotropic use, n (%)							
No	72	(41.6)	31	(43.1)	41	(56.9)	0.74
Yes	101	(58.4)	46	(45.5)	55	(54.5)	
Cord Blood Gases							
pH, Median (RIQ)	6.94	(0.11)	6.94	(0.12)	6.94	(0.11)	0.60*
HCO3, Mean (ds)	12.41	(4.90)	13.09	(4.97)	11.86	(4.80)	0.10
Excess Base, Mean (ds)	−17.91	(4.60)	−18.13	(5.01)	−17.7	(4.26)	0.56
Lactate, Mean (ds)	10.84	(4.11)	10.16	(3.90)	11.3	(4.21)	0.05

*Mann–Whitney U test as appropriate. IQR: interquartile range; SD: standard deviation.

** Minimum birth weight included in the cohort was 1935 g (newborns with birth weight <1800 g were excluded by study design).

A composite outcome of in-hospital mortality or abnormal brain MRI findings within the first week was observed in 40.6% of the patients (n = 70). Among these, 12 neonates (6.9%) died, 61 (37.2%) had abnormal MRI findings, and three patients experienced both events, accounting for the overlap between components of the composite outcome. A clinical review indicated that deaths occurred predominantly in neonates with severe hypoxic-ischemic encephalopathy and were related to neurological deterioration and/or withdrawal of life-sustaining treatment based on poor neurological prognosis.

In univariate analysis (Table 2), the timing of TH initiation (≤6 h vs. > 6 to ≤12 h) was not significantly associated with the composite outcome (p = 0.32). However, a gestational age < 37 weeks was associated with an increased risk (p = 0.01). Seizures, either clinical or electrographic, were more common among neonates with the outcome (OR, 4.1; 95% CI: 2.11–8.16; p < 0.001), and higher cord blood lactate levels were observed in this group (p = 0.002).

**Table 2:** Association of clinical and biochemical variables with the composite outcome of death or brain injury on MRI.

Variable	Death/ Brain MRI alteration				P-value
	No		Yes		
Onset of therapeutic hypothermia (hours), n (%)					
≤6	49	(63.6)	28	(36.4)	0.32
6 - 12 hours	54	(56.3)	42	(43.7)	
Gestational age (Ballard), n (%)					
≥37 weeks	96	(62.8)	57	(37.2)	**0.01**
35 - 37 weeks	7	(35.0)	13	(65.0)	
Birth weight (g), n (%)**					
≥2500	92	(61.3)	58	(38.7)	0.22
<2500	11	(47.8)	12	(52.2)	
Sex, n (%)					
Female	44	(66.7)	22	(33.3)	0.13
Male	59	(55.1)	48	(44.9)	
Admission temperature (°C), n (%)					
33 - 35	65	(67.0)	32	(33.0)	0.07
<33	13	(46.4)	15	(53.6)	
>35	25	(52.1)	23	(47.9)	
Birth resuscitation, n (%)					
No	11	(68.7)	5	(31.3)	0.43
Yes	92	(58.6)	65	(41.4)	
Severity encephalopathy (Sarnat), n (%)					
Moderate	71	(63.4)	41	(36.6)	0.16
Severe	32	(52.5)	29	(47.5)	
Asphyxia severity, n (%)					
Moderate	46	(66.7)	23	(33.3)	0.12
Severe	57	(54.8)	47	(45.2)	
APGAR 5 minutes, n (%)					
>5	54	(62.8)	32	(37.2)	0.39
≤5	49	(56.3)	38	(43.7)	
APGAR 10 minutes, n (%)					
>5	96	(61.1)	61	(38.9)	0.18
≤5	7	(43.8)	9	(56.2)	
Seizures, n (%)					
No	81	(72.3)	31	(27.7)	**<0.001**
Yes	22	(38.6)	35	(61.4)	
Inotropic use, n (%)					
No	42	(58.3)	30	(41.7)	0.78
Yes	61	(60.4)	40	(39.6)	
Cord Blood Gases					
pH, Median (RIQ)	6.93	(0.11)	6.94	(0.10)	0.59*
HCO3, Mean (ds)	12.8	(4.89)	11.9	(4.80)	0.13
Excess Base, Mean (ds)	−18.1	(5.00)	−17.7	(4.62)	0.56
Lactate, Mean (ds)	10.2	(3.90)	11.3	(4.21)	**0.05**

IQR: interquartile range; ds: standard deviation*Mann-Whitney U test.

** Minimum birth weight included in the cohort was 1935 g (newborns with birth weight <1800 g were excluded by study design).

Regarding transport-related thermal status, 56.1% of neonates arrived at admission temperatures between 33–35 °C, consistent with passive cooling, whereas 16.2% arrived at temperatures <33 °C. The admission temperature category did not differ significantly between the early and late therapeutic hypothermia groups and was not independently associated with the composite outcome.

Crude outcome frequencies stratified by HIE severity and the timing of therapeutic hypothermia initiation are provided in S1 Table for descriptive purposes.

**Fig 1:**
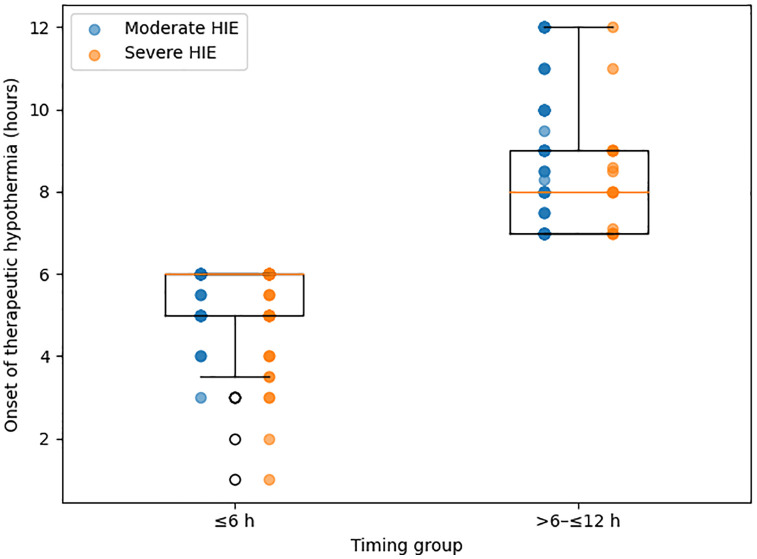
Distribution of onset of therapeutic hypothermia by timing group and HIE severity. Boxplot showing the distribution of the onset of therapeutic hypothermia (hours) across timing categories (≤6 h vs. > 6– ≤ 12 h), stratified by hypoxic–ischemic encephalopathy (HIE) severity. Individual points represent each neonate.

To complement the descriptive analysis, we plotted the continuous distribution of the variable onset of therapeutic hypothermia by timing group (≤6 h vs. > 6–12 h) and stratified by HIE severity (Fig 1). This visualization illustrates the expected separation between early and late initiation, and shows similar distributions for moderate and severe cases within each timing category.

In the multivariate logistic regression model (Table 3), delayed TH initiation was not independently associated with the composite outcome (adjusted odds ratio [aOR], 1.83; 95% CI: 0.86–3.90). In contrast, severe HIE (aOR, 2.28; 95% CI: 1.01–4.99) and seizures (aOR, 4.25; 95% CI: 2.09–8.61) remained significant predictors.

**Table 3:** Crude and adjusted odds ratios for the association between the timing of therapeutic hypothermia initiation and the composite outcome of death or brain injury on MRI.

Variable	Crude OR	95% CI		aOR	95% CI	
Onset of therapeutic hypothermia (hours)						
≤6 (reference)	1.00			1.00		
≥6 - ≤ 12 hours	1.36	0.75	2.51	1.83	0.86	3.90
Gestational Age (Ballard)						
≥37 weeks (baseline)	1.00			1.00		
< 37 weeks	3.12	1.18	8.29	2.70	0.92	7.90
Severity of encephalopathy (Sarnat)						
Moderate (reference)	1.00			1.00		
Severe	1.57	0.83	2.95	2.28	1.04	4.99
Seizures						
No (reference)	1.00			1.00		
Yes	4.16	2.11	8.16	4.25	2.09	8.61

Abbreviations: OR, odds ratio; aOR, adjusted odds ratio; CI, confidence interval.

Final multivariable logistic regression model adjusted for gestational age, severity of encephalopathy, and seizures, selected from a prespecified clinical model.

Secondary analysis was conducted using a logistic regression model restricted to neonates who underwent brain MRI, excluding those who died before imaging (n = 9). Two patients who died after the MRI were included in the analysis. In this adjusted model (Table 4), initiation of TH between 6 and 12 h was associated with a non-significant increase in the odds of MRI-confirmed brain injury compared with ≤6 h (aOR: 1.76; 95% CI: 0.81–3.86). Severe encephalopathy also showed a non-significant trend toward increased risk (aOR: 2.27; 95% CI: 0.98–5.29), while seizures remained a strong independent predictor of brain injury (aOR, 3.93; 95% CI: 1.90–8.13).

**Table 4:** Crude and adjusted odds ratios for the association between timing of therapeutic hypothermia and brain MRI-confirmed neurological injury.

Variable	Crude OR	95% CI		aOR	95% CI	
Onset of therapeutic hypothermia (hours)						
≤6 (reference)	1.00			1.00		
≥6 - ≤ 12 hours	1.22	0.64	2.31	1.76	0.81	3.86
Gestational Age (Ballard)						
≥37 weeks (reference)	1.00			1.00		
< 37 weeks	2.68	0.96	7.49	2.44	0.80	7.40
Severity of encephalopathy (Sarnat)						
Moderate (reference)	1.00			1.00		
Severe	1.25	0.64	2.44	2.27	0.98	5.29
Seizures						
No (reference)	1.00			1.00		
Yes	3.56	1.78	7.09	3.93	1.90	8.13

Abbreviations: OR, odds ratio; aOR, adjusted odds ratio; CI, confidence interval.

Multivariable logistic regression adjusted for admission temperature, gestational age, severity of encephalopathy, and seizures.

## Discussion

This study evaluated the association between the timing of therapeutic hypothermia (TH) initiation (≤6 h vs. > 6– ≤ 12 h) and adverse outcomes in neonates with moderate-to-encephalopathy (HIE). We found that delayed initiation (>6 to ≤12 h) was not significantly associated with an increased risk of composite outcomes (mortality and/or abnormal brain MRI findings) or MRI-confirmed brain injuries. In contrast, severe HIE and clinical or electrographic seizures have consistently emerged as strong independent predictors of adverse outcomes, underscoring their critical prognostic relevance in this population.

These findings should be interpreted in the context of prior evidence evaluating the efficacy of TH, which is initiated beyond the conventional six-hour therapeutic window. Unlike the randomized controlled trials (RCTs) by Laptook et al. and Laal et al., which compared late TH with no hypothermia [22,23], our study included only neonates who received TH, differing only in the timing of initiation (≤6 h vs. > 6–12 h). Laptook et al. randomized neonates to receive TH between 6 and 24 hours of life or normothermia and reported no significant reduction in neurological disability with late TH, although a trend toward benefit was noted in neonates with moderate HIE [22]. Similarly, Laal et al. compared late TH (>6 h) with standard care without cooling and found a significant reduction in early mortality but did not assess long-term neurological outcomes [23]. The observed benefits of late TH administration in these studies may partially reflect the absence of treatment in the control group. In contrast, in our cohort, in which both groups received TH, no significant differences in short-term outcomes were observed between early and delayed initiation.

Therefore, the role of HIE severity warrants careful consideration when interpreting these findings. More severe cases are often identified earlier and prioritized for prompt intervention, potentially introducing confounding factors. In our multivariable model, HIE severity remained a significant independent predictor of adverse outcomes, whereas timing did not, suggesting that neurological status at presentation may exert a stronger prognostic influence than precise timing of hypothermia initiation. This observation aligns with clinical practice and current recommendations, such as those from the *Colombian Neonatology Association (ASCON)*, which consider delayed TH (>6 h) a relative contraindication, while acknowledging that certain neonates, particularly those with moderate HIE, may still benefit from treatment [21]. ASCON recommendations are aligned with international guidelines in defining initation within 6 hours as the standar of care, consistent with ILCOR and AAP guidance; the consideration of hypothermia beyond 6 hours reflects expert consensus aimed at addressing unavoidable delays in referral and transport common in low- and middle-income settings. Supporting this interpretation, Jia et al. reported that late TH improved outcomes in neonates with moderate HIE but not in those with severe HIE [16], reinforcing the notion that the benefit of TH is modulated by the severity of encephalopathy and underscoring the importance of individualized clinical assessment rather than rigid adherence to time thresholds.

The lack of a significant association between delayed TH initiation and adverse outcomes in our study may also reflect the dynamic pathophysiology of hypoxic-ischemic brain injury that extends beyond the first few hours of life. Secondary energy failure, characterized by oxidative stress, mitochondrial dysfunction, and activation of inflammatory cascades, typically occurs between 6 and 24 h after insult, and contributes to neuronal death through necrosis and apoptosis [24–26]. This window suggests that some neonates, particularly those with moderate HIE, may benefit from treatment initiated after 6 h. Conversely, in severe HIE, the rapid progression of irreversible neuronal injury may limit the efficacy of delayed hypothermia. These mechanisms may explain the absence of significant differences between early and late TH observed in our study, and highlight the need for further research on the biological underpinnings of neuroprotection.

Our findings contribute to the ongoing debate regarding the optimal therapeutic window for hypothermia, particularly in low- and middle-income countries (LMICs) where delays in referral and access to specialized care often hinder timely treatment initiation. These insights may inform strategies aimed at optimizing implementation in resource-limited settings.

Neonatal transport remains a major challenge in LMICs, with up to 67% of neonates with HIE requiring referral to higher-level centers [27]. Previous studies have shown that outborn neonates experience worse neurological outcomes, likely due to inadequate initial stabilization and exposure to uncontrolled hypothermia before hospital admission [28]. These factors may have contributed to the lack of significant differences observed between early and late TH initiation in the present study.

Consistent with global evidence, TH should ideally be initiated within 6 hours of birth to maximize neuroprotection. However, in our context, initiation beyond six hours remains a common practice. These findings highlight the urgent need to strengthen early identification and referral systems to minimize delays in treatment initiation. In Colombia, most neonatal transfers to high-complexity units originate from low- and medium-complexity healthcare institutions and are largely unregulated, accounting for approximately 96% of referrals [29]. Moreover, in other low-resource settings, up to 25% of neonates arrive at referral facilities more than 24 hours after birth, with an average age at arrival of 18 hours [30]. Birth asphyxia was the most common reason for referral, accounting for 37% of cases [30]. These systemic delays represent a critical gap in perinatal care that directly undermines the timely initiation and potential effectiveness of TH.

These findings should not be interpreted as endorsing delayed initiation of therapeutic hypothermia. Initiation within the first 6 hours remains the recommended standard of care globally; however, our results reflect the reality of unavoidable delays in LMIC referral systems, where starting TH between 6 and 12 hours may still be reasonable in selected cases but should never replace efforts to achieve timely initiation.

In LMIC settings, the external validity of our results must be interpreted within the broader structural constraints that shape neonatal care. Systematic evidence demonstrates that delays in the recognition of neonatal illnesses, fragmented referral pathways, and uneven access to higher-level facilities are pervasive barriers that influence newborns’ definitive care [31]. Likewise, quality improvement analyses highlight that outcomes for infants with HIE often depend not only on clinical management but also on human, systemic, and environmental factors, such as variability in team expertise, transport coordination, and initial stabilization practices across facilities [32]. Global reviews have also underscored substantial intercenter variability in the implementation of TH, even in high-resource settings, emphasizing that structural and operational factors strongly modulate outcomes [33]. These shared systemic challenges suggest that our findings may be applicable to other LMICs where delays in referral and heterogeneity in perinatal care remain common.

Seizures also emerged as independent predictors of mortality and/or brain injury on MRI in our cohort, consistent with prior reports identifying them as markers of disease severity and potential contributors to secondary brain injury [34,35]. This finding underscores the importance of the early detection and management of seizures to improve neurological outcomes in neonates with HIE.

This study has several methodological strengths. First, by focusing exclusively on neonates who received servo-controlled TH, we minimized the variability in exposure and ensured uniform adherence to standard cooling protocols, which has rarely been reported in observational studies. Second, the use of brain MRI within the first week of life as an objective and sensitive marker of neurological injury provided robust and clinically meaningful outcome data. Finally, the analysis was conducted in a real-world setting within an LMIC, reflecting the challenges of neonatal transport and the delayed referral inherent in such contexts, thereby improving the generalizability of our findings to similar environments.

Nevertheless, this study has limitations inherent to its retrospective observational design, including potential selection and information biases, particularly regarding the preadmission clinical data. Detailed transport metrics (e.g., referral distance or time-to-referral) were not systematically available, limiting the ability to empirically disentangle logistical from clinical drivers of hypothermia timing. Conducting the study at a single tertiary referral center in a lower-middle-income country may limit the generalizability of the findings to settings with different levels of neonatal care and transport systems. Although multivariable models were adjusted for key confounders, unmeasured variables, such as quality of transport, adequacy of ventilatory support, and hemodynamic status upon admission, may have influenced the outcomes. In our context, most neonates are identified at birth in level I–II facilities and referred immediately. Therefore, delays in initiating therapeutic hypothermia are driven largely by transport availability and system-level factors rather than clinical discretion. This structure may partially mitigate, but does not eliminate, the risk of indication bias.

Additionally, the study was not powered to detect differential effects across HIE severity levels and exploratory stratified analyses yielded imprecise and clinically inconsistent estimates. Therefore, we relied on a primary-adjusted model for interpretation.

Future research using prospective multicenter cohorts or randomized designs is critical to characterize the effects of delayed TH initiation more definitively, particularly in relation to HIE severity. Additionally, integrating biomarkers of neuronal injury and long-term neurodevelopmental follow-up would allow for a more comprehensive assessment of the efficacy and safety across an extended therapeutic window.

In conclusion, this study found no significant differences in mortality or MRI-confirmed brain injury between neonates with moderate or severe perinatal asphyxia who received TH initiated early (<6 h) or late (6–12 h) after birth. In multivariable analysis, seizures and severe HIE emerged as independent predictors of neurological injury and/or neonatal death. These findings underscore the need for further research to elucidate the potential benefits of delayed TH, particularly in neonates with moderate HIE, and to better understand how disease severity and other clinical factors influence the optimal timing of TH in this high-risk population.

Importantly, this study did not establish non-inferiority or equivalence between the early and delayed initiation of therapeutic hypothermia. Given its observational design and limited statistical power, these findings should not be interpreted as supporting delayed therapeutic hypothermia, and initiation within the first 6 h of life remains the recommended standard of care whenever feasible.

## Supporting information

S1 Table Crude outcome frequencies stratified by HIE severity and the timing of therapeutic hypothermia initiation. (DOCX)
