# Supplementary material for: Timing of therapeutic hypothermia and outcomes in neonates with hypoxic-ischemic encephalopathy: A cohort study in a middle-income country
Source: PLoS One. 2026 Mar 9;21(3):e0343589. doi: 10.1371/journal.pone.0343589 (PMC12970932; doi:10.1371/journal.pone.0343589)
Supplement: S1 Table — (DOCX) [file pone.0343589.s001.docx]

S1 Table

Crude rates of the composite outcome stratified by HIE severity and timing of therapeutic hypothermia initiation

| **HIE severity** | **TH initiation time** | **No outcome n (%)** | **Composite outcome n (%)** | **Total (n)** |
| --- | --- | --- | --- | --- |
| Moderate | ≤6 h | 27 | 7 | 34 |
| Moderate | >6–≤12 h | 44 | 34 | 78 |
| Severe | ≤6 h | 22 | 21 | 43 |
| Severe | >6–≤12 h | 10 | 8 | 18 |
| This table presents descriptive outcome frequencies only. No statistical comparisons were performed due to limited sample size within strata. | | | | |
